# First molecular evidence of hepatitis E virus in farmed raccoon dogs

**DOI:** 10.1080/22221751.2024.2361025

**Published:** 2024-05-27

**Authors:** Fengjuan Tian, Jing Li, Yang Liu, Wenli Liu, Yue Liu, Shan Xu, Yigang Tong, Fumin Feng

**Affiliations:** a Beijing Advanced Innovation Center for Soft Matter Science and Engineering, College of Life Science and Technology, Beijing University of Chemical Technology, Beijing, People’s Republic of China; b School of Public Health, North China University of Science and Technology, Tangshan, People’s Republic of China; c Institute of Analysis and Testing, Beijing Academy of Science and Technology (Beijing Center for Physical and Chemical Analysis), Beijing, People’s Republic of China

**Dear editor,**

Raccoon dog (*Nyctereutes procyonoides*) is a small, heavy-set, fox-like canid with fur markings similar to those of raccoons (*Procyon lotor*). The fur of raccoon dogs is dense and soft, contributing to its widespread breeding and commercial trade as a fur-bearing species in China in recent decades. They are omnivores that feed on insects, rodents, amphibians, birds, fish, reptiles, mollusks, human garbage, carrion, and eggs, as well as fruits, nuts, and berries [1]. Therefore, their living and feeding habits provide the potential link to both mammal viruses and arthropod-associated viruses. Raccoon dogs are the host of many zoonotic viruses, such as rabies viruses, coronaviruses, and rotaviruses [2,3]. As an important reservoir for zoonotic viruses, raccoon dogs harbour various potentially high-risk pathogens, although their role as direct hosts for the transmission of these viruses to humans is often uncertain, it still highlights the importance of raccoon dogs as potential drivers of disease emergence in spillover and spillback.

Hepatitis E virus (HEV) is a quasi-enveloped single-stranded positive-sense RNA virus belonging to the subfamily *Orthohepevirinae* in the family *Hepeviridae*. According to the International Committee on the Taxonomy of Viruses (ICTV), the subfamily *Orthohepevirinae* is composed of four genera: *Avihepevirus* (birds), *Chirohepevirus* (bats), *Paslahepevirus* (mammals) and *Rocahepevirus* (rodents). While *Chirohepevirus* is being reconsidered as an independent genus within the family *Hepeviridae*, taxonomically [4]. The latter two genera are considered a potential zoonotic threat to humans and animals [5,6]. Among those species affecting humans, *Paslahepevirus balanyani*, is a leading cause of acute viral hepatitis worldwide. Every year there are an estimated 20 million HEV infections worldwide, leading to an estimated 3.3 million symptomatic cases of hepatitis E (https://www.who.int/news-room/fact-sheets/detail/hepatitis-e). In total, there are eight genotypes of HEV. HEV-1, HEV-2, HEV-3, and HEV-4 are usually linked to human HEV infections. Genotypes HEV-1 and HEV-2 are restricted to humans, whereas genotypes HEV-3 and HEV-4 exhibit a wider host range and become the cause of most zoonotic infections, encompassing species such as rat, deer, mongoose, rabbit, moose, bottlenose dolphin, sheep and cattle [7]. Pigs serve as the principal reservoir for genotypes HEV-3, HEV-4, while HEV-5 and HEV-6 have only been detected in wild boar in Japan [8]. Genotypes HEV-7 and HEV-8 are predominantly identified within the camel population [7]. Recently, serological and virological investigations for HEV in both domestic dogs and raccoon dogs were performed and the results indicated the presence of anti-HEV antibodies. However, neither HEV RNA [9–11] nor signs of hepatic damage were found [12]. Of note, contact with dogs has been identified as a risk factor for anti-HEV IgG positivity in human population [13]. Previous studies have suggested that canids may serve as a transmission link for HEV [14]. In China, given the economic significance of raccoon dogs among canids in farming, it is imperative to conduct investigations on HEV in raccoon dogs. In this study, an epidemiological survey was performed in raccoon dogs to determine the molecular prevalence of HEV in northern China between 2022 and 2023, confirming the natural susceptibility of this species to HEV infection. We first reported 15 nearly complete sequences for evolutionary analysis of HEV harboured by raccoon dogs. We have provided evidence of HEV circulating in raccoon dogs, suggesting that raccoon dogs may serve as a new route for HEV transmission. This study provides crucial insights into the molecular prevalence, genome characteristics, genetic diversity, and zoonotic potential of HEV in raccoon dogs.

From 2022 to 2023, we collected 100 faecal samples from 100 healthy raccoon dogs and eight tissue samples from two deceased raccoon dogs from different fur animal farms in Hebei province, China, for detecting the presence of HEV and gathering its genomic information (Supplementary Table 1). Tissue samples consisting of kidneys, livers, intestines, and lungs were homogenized with steel beads. Then, all samples were centrifuged at 12,000 × g for 3 min at 4 °C to obtain supernatant for viral RNA extraction using an Omega Viral DNA/RNA Kit (Omega, GA, USA). All samples were detected using broadly reactive HEV detection primers and a probe by real-time quantitative reverse transcription PCR (RT-qPCR) individually, and the prevalence of HEV RNA was 23.5% (24 positive from 102 donors) (Supplementary Table 1 and Table 2). The samples that tested positive for HEV by RT–PCR were further processed for NGS, including kidney, liver, lung, and intestinal samples from two deceased raccoon dogs, as well as 22 faecal samples from 22 healthy raccoon dogs. The RNA from eight tissue samples were organized into two pools based on different donors and 22 faecal samples underwent individual NGS analysis. In total, 24 libraries were constructed using the NEBNext Ultra II Directional RNA Library Prep Kit for Illumina (NEB, MA, USA). Finally, the prepared libraries were sequenced on the Illumina NovaSeq 6000 (PE150) sequencing platform (Illumina, San Diego, USA). Sequencing reads were first quality controlled using fastp (v0.20.0) [15]. The remaining quality reads were assembled de novo using SPAdes (v3.13.0). The assembled contigs of HEV were confirmed by comparing against the NCBI non-redundant protein database (nr) using Diamond blastx [16]. For incomplete viral genomes, we then designed specific primers according to assembled HEV contigs. And then the internal gaps were filled by RT–PCR and Sanger sequencing. 15 complete or nearly complete HEV genomes were obtained and a read-mapping approach was used to estimate the depth of confirmed HEV (Figure 1(A)).

**Figure 1.**
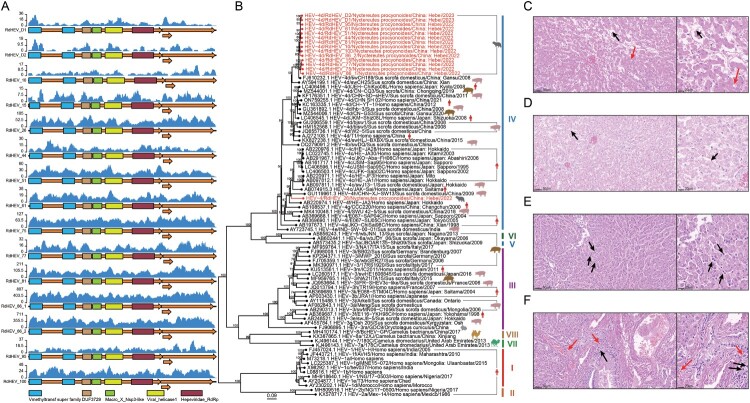
(A) Schematic representation of the HEV genome sequences obtained in this study, along with reads mapping to the acquired HEV genome. (B) Phylogenetic analysis of near-complete HEV sequences harboured by raccoon dogs. The subtypes of sequences were determined using the Hepatitis E Virus Genotyping Tool (https://www.rivm.nl/mpf/typingtool/hev/). The phylogeny was constructed using the maximum likelihood method with the best model inferred by IQ-TREE2, based on nucleic acid sequences of species *Paslahepevirus balayani*. The scale bar represents the evolutionary divergence distance inferred by the number of nucleotide substitutions per site. The numbers at each node indicate bootstrap probabilities determined from 1000 replicates. HEV sequences obtained in this study are highlighted in red lettering. (C and D) Histological examination (HE) of the liver from Raccoon dog D1. Black arrows in panel C indicate localized hepatic cord rupture, and red arrows indicate disrupted hepatic cell structure. Black arrows in panel D indicate hepatocyte vacuolization. (E and F) HE staining of liver tissue from Raccoon dog D2. Black arrows in panels E and F indicate macrophages, and red arrows in panel F indicate lymphocyte infiltration.

The genomic sequences of HEV obtained in this study, along with representative *Paslahepevirus balayani*, were aligned using MAFFT. Phylogenetic analysis was then performed using the maximum likelihood method using IQ-TREE 2 [17]. Our findings revealed that HEVs isolated from raccoon dogs diverged into two genetic lineages within the HEV-4 subtype (Figure 1(B)). One branch consists of 14 sequences that are most closely related to HEV-4d. These sequences share 95.22–96.22% nucleic acid sequence identity with the swine HEV isolate swCH189. The other branch contains only RdHEV_26, forming a separate branch that is significantly distant from known genotypes. RdHEV_26 is most closely related to the human HEV isolate HE-JA1, with a nucleic acid homology of 86.66%. We also observed differences among the sequences we discovered. RdHEV_26 shows significant divergence from other sequences, with a homology of only around 83%. The homology between the other sequences ranges from 93.96% to 99.74% (Supplementary Table 3). To observe the presence of liver damage in raccoon dogs due to HEV, we conducted pathological examinations on the liver tissues of two deceased raccoon dogs (D1, D2). The tissue sample of liver was fixed with 10% formalin solution for 24 h, embedded in paraffin, sectioned at 4–6 μm, and stained with hematoxylin–eosin followed by microscopic observation (Nikon ECLIPSE TS100). We observed no severe lesions in livers, only localized disruption of hepatic cell structure (Figure 1(C)), hepatocyte vacuolization (Figure 1(D)), a small number of macrophages (Figure 1(E)), and lymphocyte infiltration (Figure 1(F)), among other symptoms. Additionally, we conducted Immunohistochemistry testing, unfortunately, we were unable to localize HEV particles. We speculate that this may be attributed to existing antibodies’ inability to specifically bind to the newly discovered HEV in raccoon dogs. Consequently, we lack further evidence to establish the association between liver symptoms and HEV.

## Discussion

This study presents molecular characterization and phylogenetic analysis of HEV-4 in farmed raccoon dogs. Complete HEV genomic sequences from diverse parts and species globally are crucial for further understanding the HEV-4 circulation patterns in both human and animal populations. Our discovery represents the first molecular evidence demonstrating raccoon dogs as reservoir hosts for HEV. Furthermore, the positivity rate of HEV among healthy raccoon dog faecal samples in this study was 22%, indicating a widespread and long-term circulation of HEV in healthy raccoon dogs. Phylogenetic analysis revealed that the HEV isolated from raccoon dogs formed two branches within the HEV-4 subtype, with one branch sharing the highest similarity with swine HEV isolate swCH189 and the other with Human HEV isolate HE-JA1, the latter showing genetic differences from known HEV-4 subtypes. This discovery enhances our understanding of HEV genetic diversity and demonstrates the susceptibility of raccoon dogs to multiple HEV subtypes. The principal mode of HEV transmission is through the faecal-oral route. Therefore, it is crucial to handle livestock faeces responsibly to prevent water source contamination and mitigate potentially severe consequences.

In conclusion, this report provides important insights into the molecular prevalence, evolutionary dynamics, liver pathology and zoonosis risk of HEV in raccoon dogs. Identifying viruses with the potential to emerge in humans or spillover into other animal populations aids pandemic preparedness. Our study has certain limitations: we cannot prove the contribution of HEV to liver damage in raccoon dogs. However, the detection of HEV in faecal samples from healthy raccoon dogs highlights the zoonotic disease risk in the raccoon dog farming industry. The identified HEVs are closely related to those in domestic pigs, wild boar and humans. Although the transmission route of HEV among them remains uncertain, farmers, abattoir workers, handlers and other relevant professionals in the animal industry should strengthen the detection and prevention of HEV, reducing the risk of HEV outbreaks from animal sources.

## Supplementary Material

Supplemental Material

Supplemental Material

Supplemental Material

## Data Availability

The data reported in this paper have been deposited in the GenBase in National Genomics Data Center, Beijing Institute of Genomics, Chinese Academy of Sciences/China National Center for Bioinformation, with the accession number C_AA067530.1-C_AA067544.1, which is publicly accessible at https://ngdc.cncb.ac.cn/genbase.
