# Supplementary material for: First molecular evidence of hepatitis E virus in farmed raccoon dogs
Source: Emerg Microbes Infect. 2024 May 27;13(1):2361025. doi: 10.1080/22221751.2024.2361025 (PMC11177704; doi:10.1080/22221751.2024.2361025)
Supplement: Supplemental Material [file TEMI_A_2361025_SM9998.docx]

Supplementary Table2. The primers of RT-qPCR used according to the paper.

| Primer or Probe | Nucleotide sequence (5'-3') | Reference |
| --- | --- | --- |
| JVHEVF | RGTGGTTTCTGGGGTGAC | [1] |
| JVHEVR | AGGGGTTGGTTGGATGAA |  |
| JVHEVP | 5'-6-FAM-TGATTCTCAGCCCTTCGC-BHQ-3' |  |

Reference

1. Jothikumar N, Cromeans TL, Robertson BH, et al. A broadly reactive one-step real-time RT-PCR assay for rapid and sensitive detection of hepatitis E virus. J Virol Methods. 2006 Jan;131(1):65-71.
